# Supplementary figures and images for: Socioeconomic disparities and sexual dimorphism in neurotoxic effects of ambient fine particles on youth IQ: A longitudinal analysis
Source: PLoS One. 2017 Dec 5;12(12):e0188731. doi: 10.1371/journal.pone.0188731 (PMC5716576; doi:10.1371/journal.pone.0188731)

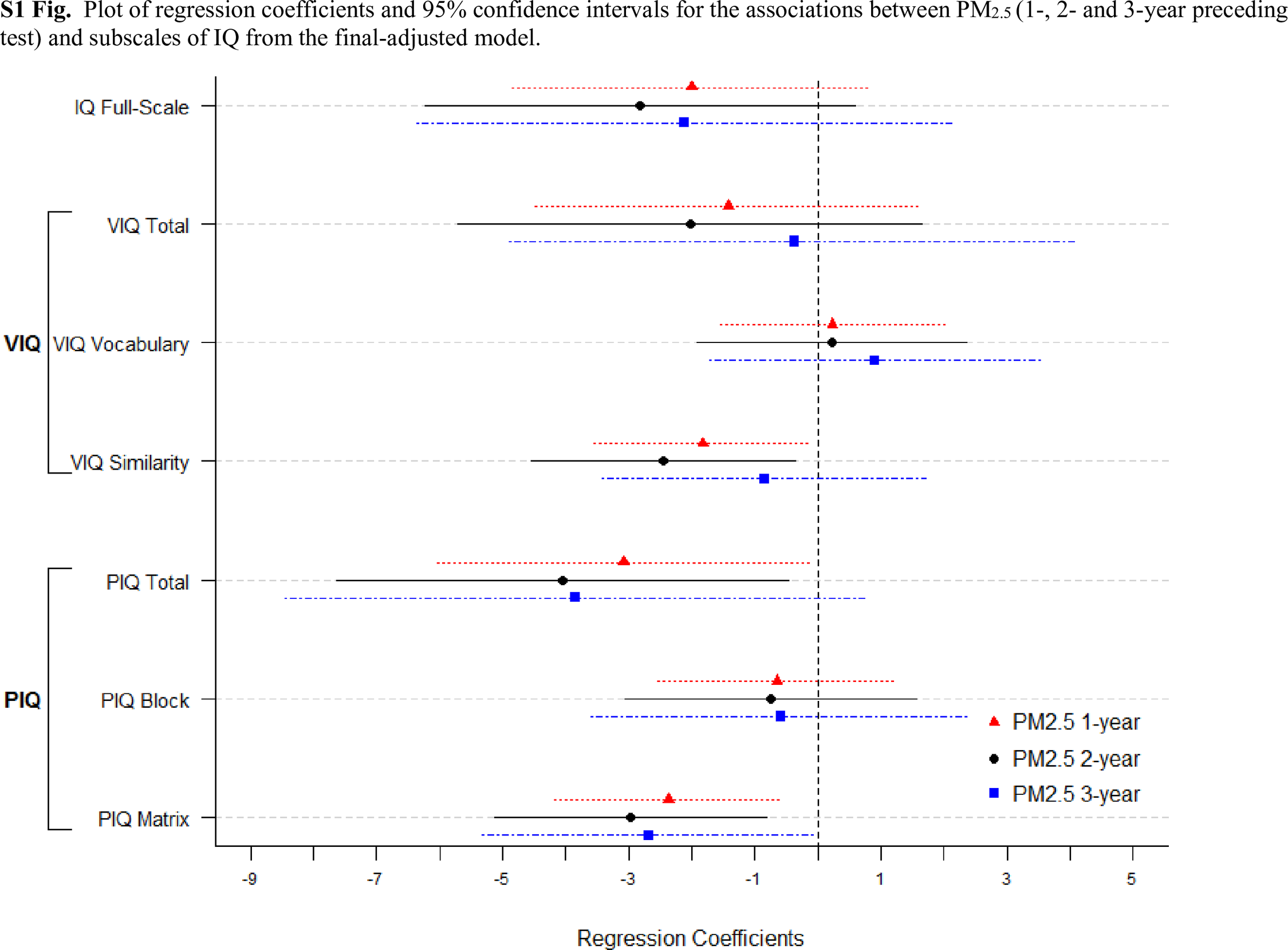

Supplement: S1 Fig — (TIF) [file pone.0188731.s002.tif]
