# Supplementary material for: Socioeconomic disparities and sexual dimorphism in neurotoxic effects of ambient fine particles on youth IQ: A longitudinal analysis
Source: PLoS One. 2017 Dec 5;12(12):e0188731. doi: 10.1371/journal.pone.0188731 (PMC5716576; doi:10.1371/journal.pone.0188731)
Supplement: S1 File — A. Map of Residential Locations during pre-/early- adolescence and emerging adulthood; B. Temporal-spatial Modeling of PM2.5 Exposure; C. Relevant Covariates; D. Summary Table of Air Pollution and IQ Studies. (PDF) [file pone.0188731.s003.pdf]

S1 File. Appendix

A. Map of Residential Locations during pre-/early- adolescence and emerging adulthood

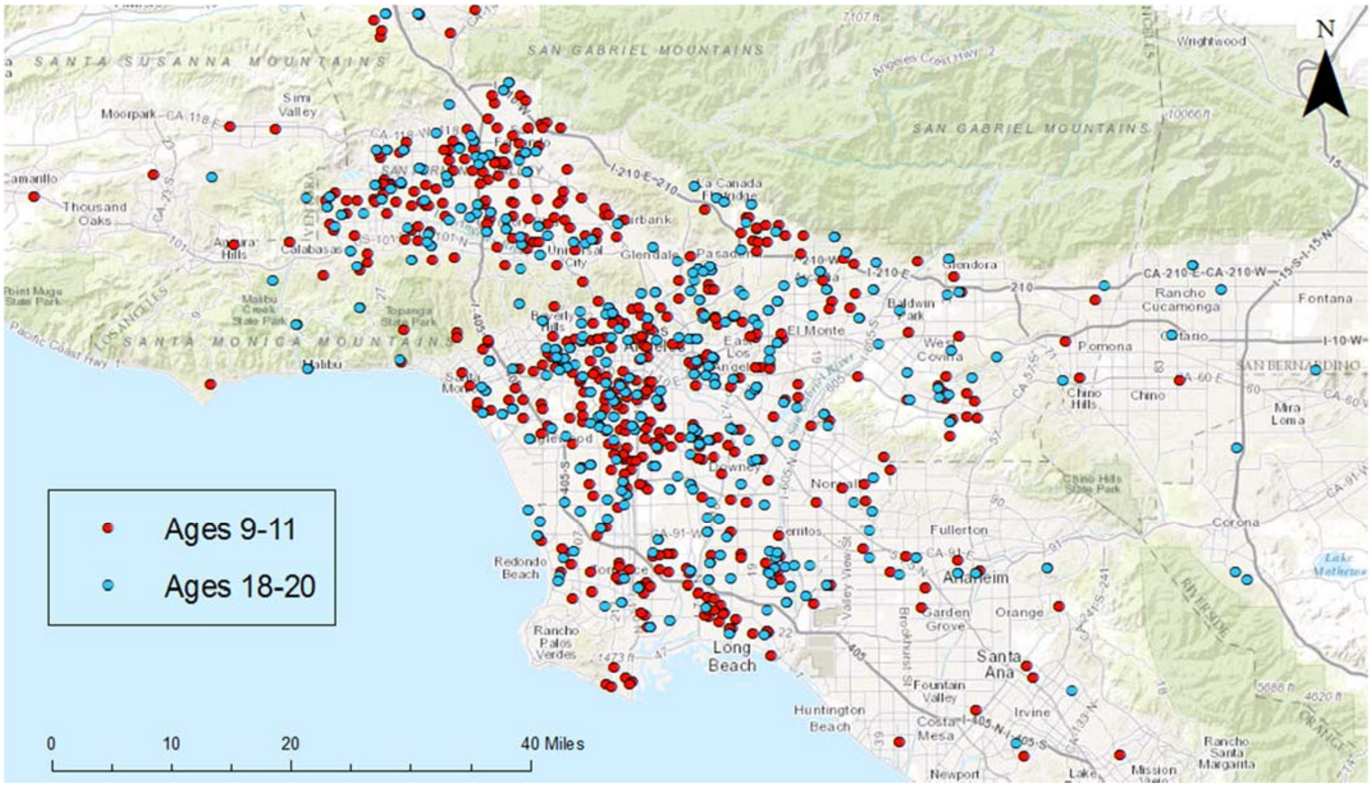

B. Temporal-Spatial Modeling of PM2.5 Exposure

Daily air pollution concentrations for particulate matter with aerodynamic diameter less than 2.5 μm ($PM_{2.5}$) were acquired from the US Environmental Protection Agency's Technology Transfer Network (1) for the years 2000 through 2014. For exposure model development, we aggregated the daily concentrations from the monitoring sites located in our study domain to monthly averages. A generalized additive model (GAM) (2) was fit to generate exposure estimates at subjects' residences. The GAM is an extension of linear regression whereby spline basis functions are used to allow for smooth (non-linear) relationships between the predictor and response variables. To predict monthly $PM_{2.5}$ at the subject locations, we incorporated smooth functions of space and time in the model

$$[PM_{2.5}]_{s,t}= \alpha+ f_s(x,y) + f_t(t)+\varepsilon_{s,t} \qquad \text{(Eq.1)}$$

where $[PM_{2.5}]_{s,t}$ is the $PM_{2.5}$ concentration (μg/m^3), α is the intercept, $f_s(x,y)$ is a 2-dimensional thin plate spline for space, s, referenced by geographic location x and y, $f_t(t)$ is a cubic regression spline for time, t, referenced by calendar month, and $\varepsilon_{s,t}$ is the iid $N(0,\sigma^2)$ residual error.

Including only those sites that met a 75% data completeness criterion, there were 25 monitoring locations in our study area with which we developed the spatiotemporal GAM exposure model. Initial model diagnostics indicated that shorter five-year GAM models generated more accurate exposure predictions than modeling the full time series in one model. Furthermore, to alleviate problems predicting on the boundaries of the time series, we split up the data into five-year segments with overlapping years (i.e. 2000-2005, 2004-2008, 2007-2011, 2010-2014) and fit separate GAM models to each segment. The average model R^2 for the 5 five-year GAM models was 0.71. The appropriate segmented model was then used to predict $PM_{2.5}$ concentrations at the geocoded home locations of the subjects. A monthly time-series of $PM_{2.5}$ data from 2000-2014 was created and the monthly estimates were aggregated to represent the $PM_{2.5}$ estimates 1-, 2-, and 3-years preceding baseline (i.e., the first valid IQ assessment), as well as the cumulative exposure over follow-up. These different exposure

estimates allow us to examine whether the putative effect might have started before and continued into the adolescence.

C. Relevant Covariates

Covariates were considered as potential confounders if they were known to predict IQ and likely influence where people lived (and thus their exposure to $PM_{2.5}$). These included age, gender, race/ethnicity, family SES, parents' cognitive abilities, parent-reported neighborhood quality, neighborhood SES, traffic density and neighborhood greenness. Other spatial covariates, including the CALINE-estimated total annual nitrogen oxides (NO_x) and temperature/humidity, are yet unknown with regard to their associations with IQ. Parent-level risk factors (operationalized as maternal smoking during pregnancy and parental perceived stress) may relate to IQ development but contribute little to people's residence. The potential confounding by total annual NO_x, temperature/humidity, and parent-level risk factors were thus examined in the sensitivity analyses. Neighborhood greenness, traffic density and CALINE NO_x were assessed as time-varying covariates at each IQ assessment.

Family Socioeconomic Status (SES) was assessed with the Hollingshead Index based on parents' education levels, occupational status, marital status and family income (3), with higher scores corresponding to higher SES levels (range: 14 ~ 64.5).

Perceived Neighborhood Quality was assessed with a parent-reported questionnaire specifically developed for the RFAB study and includes 17 items related to criminal and gang related activities, unemployment, vandalism, and substance use that occurs in the participants' local area (4). A sum score was created, with higher scores representing a more negative perception of neighborhood quality.

Maternal Smoking during Pregnancy was used as an indicator of parental-level risk factors. Mothers were administered a maternal health questionnaire designed for the RFAB study, asking mothers if they had smoked cigarettes during their pregnancy with the twins (yes/no).

Parental Cognitive Abilities was operationalized as Woodcock Johnson reading score in the current study: Letter-Word Identification - naming letters and reading words aloud from a list, and Word Attack - to test phonetic word attack skills (5).

Parental Stress was assessed with parent reports of 13 items designed to tap how unpredictable, uncontrollable, and overloaded parents found their lives (6). Each item was rated on a five point scale, and a continuous raw score was created by summing across items, whereby higher scores indicated more stress. This questionnaire was administered during pre-/early- adolescence with a relatively high internal consistency (average Cronbach's Alpha across waves: $\alpha = 0.85$).

Meteorological Factors: Information on ambient temperature and relative humidity was obtained from the California Air Resources Board Air Quality and Meteorological Information System. Meteorological information recorded at the nearest site was assigned to each geocoded residence to create a monthly time-series of average ambient temperature (°C) and relative humidity (%) from 1990 to 2012. Temperature and relative humidity were then averaged for the periods 1-, 2-, and 3-years preceding IQ assessment.

Neighborhood Socioeconomic Status (nSES) was defined using the US Census data (7). An index at the census tract level was created by summing and standardizing (mean=0; SD=1) the following six variables obtained from the 2000 Census: (1) % of adults 25 years old with less than a high school education; (2) % of unemployed males; (3) median household income; (4) % of households with income below the poverty line; (5) % of households receiving public assistance; and (6) % of households with children that are headed by a female. An index score greater than 0 indicates tracts above the average nSES characteristics (range: -1.50 – 4.06).

Neighborhood greenness was estimated by the Normalized Difference Vegetation Index (NDVI), derived from MODerate-resolution Imaging Spectroradiometer (MODIS) at 250-meter resolution. A 16-day time-series data in 2000-2012 was gathered from the Global Agriculture Monitoring Project (8), and the normalized NDVI (0-1, with higher values indicating denser vegetation) was aggregated in

1000-m buffers surrounding residences and over various temporal scales (1-, 2-, and 3-years) preceding IQ assessment.

Traffic density was used as a proxy for noise exposure from urban traffic. Yearly average daily traffic volumes, obtained from California Department of Transportation and TeleAtlas/GDT, were allocated to roadways and used in GIS to map traffic density within 150- and 300-m radius buffers. Yearly traffic density was assigned using 2002 and 2012 roadways and average traffic volumes at each wave, respectively.

CALINE Total Annual NO_x. A CALINE4 Gaussian traffic line-source dispersion model (9) was fit to generate NO_x exposure estimates at participants' residences. The dispersion model incorporates the residence locations, roadway geometry, vehicle traffic volume and emission rate by roadway link, and meteorological conditions as model inputs. CALINE-estimated annual average ambient concentrations of NO_x from local (within 5 km) traffic were obtained at each residence for the year of each subject's testing date. The CALINE-estimated NO_x acts as an indicator of primary air pollutant emissions from local traffic.

References

1. Environmental Protection Agency. AirDataJuly 8, 2015. Available from: http://www.epa.gov/ttn/airs/airsaqs/detaildata/downloadaqsdata.htm.
2. Wood S. Generalized additive models: an introduction with R: CRC press; 2006.
3. Hollingshead AB. Hollingshead Four-Factor Index of Socio-Economic Status. New Haven, CT: Yale University; 1979.
4. Baker LA, Tuvblad C, Wang P, Gomez K, Bezdjian S, Niv S, et al. The Southern California Twin Register at the University of Southern California: III. Twin Research and Human Genetics. 2013;16(01):336-43.
5. McGrew KS, Woodcock RW, Schrank KA. Woodcock-Johnson III normative update technical manual. Itasca: Riverside Pub.; 2007.
6. Loeber R, Stouthamer-Loeber M, Van Kammen W, Farrington DP. Initiation, escalation and desistance in juvenile offending and their correlates. J Crim L & Criminology. 1991;82:36.
7. Dubowitz T, Heron M, Bird CE, Lurie N, Finch BK, Basurto-Dávila R, et al. Neighborhood socioeconomic status and fruit and vegetable intake among whites, blacks, and Mexican Americans in the United States. The American journal of clinical nutrition. 2008;87(6):1883-91.
8. NASA/GSFC UF, SSAI, UMD Dpeartment of Geography. GLAM-Global Agricultural Monitoring 2015 [Available from: http://pekko.geog.umd.edu/usda/beta/data new.php?dsRegionId=62.
9. Benson PE. A review of the development and application of the CALINE3 and 4 models. Atmospheric Environment Part B Urban Atmosphere. 1992;26(3):379-90.

D. Summary Table of Air Pollution and IQ Studies

References/ Study Design	Population	Exposure	Outcome	Main Findings (95% CI)	Potential Confounders Adjusted/ Controlled
Suglia et al. (2008) (1)<br><br>Retrospective	202 children (average 9.7±1.7 years) of pregnant women from the Maternal-Infant Smoking Study of East Boston (MISSEB) who received prenatal care (<20th week of gestation) at an urban community health center in Boston March 1986-October 1992; 57% spoke Spanish as primary language; 82% maternal education ≤high school	Average lifetime exposure determined by averaging 2,127 residential exposure day estimates of black carbon (BC) from >80 locations using a spatiotemporal LUR model to estimate 24-hour BC measures estimated at the child's birth address<br><br>Ambient source of pollutant: traffic emissions	Kaufman Brief Intelligence Test (K-BIT) administered at 8-11 years of age	<u>Effects of BC (per interquartile-range [0.4-μg/m^3] increase in log black carbon level) on K-BIT (adjusted for demographic factors vs. fully adjusted)</u><br>Vocabulary: β = -2.0 (-5.3, 1.3) vs. β = -2.2 (-5.5, 1.1)<br>Matrices: β = -4.2 (-7.7, -0.7)* vs. β = -4.0 (-7.6, -0.5)*<br>Composite: β = -3.4 (-6.6, -0.3)* vs. β = -3.4 (-6.6, -0.3)*	Demographics factors: child's age, sex, primary language spoken at home,<br>Maternal education.<br>Fully adjusted: demographic factors + in-utero and postnatal secondhand smoke exposure + birth weight + blood lead level
Perera et al. (2009) (2)<br><br>Prospective	249 children of nonsmoking Dominican and African-American women (18-35 years old) residing in Washington Heights, Harlem, or the South Bronx in NY from the Columbia Center for Children's Environmental Health (CCCEH) cohort were recruited 1998-2003 through local prenatal care clinics, and monitored in utero until 5 years old;	Personal air monitors to measure 8 airborne PAHs (benz[*a*]anthracene, chrysene, benzo[*b*]fluroanthene, benzo[*k*]fluroanthene, benzo[*a*]pyrene, indeno[1,2,3-*cd*]pyrene, disbenz[*a,h*]anthracene, and benzo[*g,h,i*]perylene) and determine maternal exposures at single time points during 3rd trimester<br>Ambient source of pollutants: traffic emissions	Wechsler Preschool and Primary Scale of Intelligence-Revised (WPPSI-R) administered at 5 years of age	<u>Effects of prenatal PAH (high: > median [2.26 ng/m^3] vs. low: < median) exposure on IQ</u><br>Full-Scale IQ: β = -4.307* (p=0.007)<br>Verbal IQ: β = -4.668* (p=0.003)<br>Performance IQ: β = -2.369 (p=0.170)	Child's sex, gestational age, ethnicity; Maternal intelligence, years of education when child was 5, ETS exposure during pregnancy, quality of the early home caretaking environment

	57% Black/43% Dominican; 62% maternal high school education				
Edwards et al. (2010) (3)<br><br>Prospective	214 children of healthy, nonsmoking pregnant women ≥ 18 years old enrolled 2001-2006 in Krakow, Poland; children reached 5 years of age by August 2009; 100% Caucasian; 91% maternal high school education	Personal air monitors to measure 8 airborne PAHs (benz[*a*]anthracene, chrysene, benzo[*b*]fluroanthene, benzo[*k*]fluroanthene, benzo[*a*]pyrene, indeno[1,2,3-*cd*]pyrene, disbenz[*a,h*]anthracene, and benzo[*g,h,i*]perylene) and determine maternal exposures over 48-hr period during 2nd or 3rd trimester<br>Ambient source of pollutants: traffic and industrial/residential coal burning emissions	Raven Coloured Progressive Matrices (RCPM) administered at 5 years of age	<u>Effects of prenatal PAHs exposure on RCPM score</u><br>High (>median [17.96 ng/m^3]) vs. Low (<median): β = -1.36 (-2.48, -0.23)*<br>Ln(PAH): β = -0.56 (-1.00, -0.11)*	Child's sex; Maternal education; Prenatal ETS in the home. After further including maternal intelligence, the betas and *p*-values for PAH both high/low and Ln-transformed were similar and significant.
Perera et al. (2012) (4)<br><br>Prospective	100 children born to nonsmoking Chinese women, ≥ 20 years old, residing within 2.5km of power plant, who gave birth at any of three Tongliang county hospitals March 4, 2002-June 19, 2002; children were followed until 5 years of age; 100% Chinese; 40% maternal education <high school	Collected umbilical cord blood at time of delivery and analyzed using HPLC/fluorescence methods to measure B[a]P-DNA adducts (proxy for PAH-DNA adducts)<br>Ambient source of pollutants: power plant emissions	Shanghai version of the WPPSI-R administered at 5 years of age	<u>Effects of PAH-DNA adducts (log-transformed) on WPSSI-R subscales</u><br>Full-Scale IQ: β = -2.42 (-7.96, 3.13)<br>Verbal IQ: β = -1.79 (-7.61, 4.03)<br>Performance IQ: β = -2.57 (-8.92, 3.79)	Child's sex and gestational age; Maternal age and education; Cord lead (log transformed)
Lovasi et al. (2014) (5)<br><br>Prospective	326 children born between 1998 and 2006 to nonsmoking Dominican and African-American	Personal air monitors to measure 8 airborne PAHs (benz[*a*]anthracene, chrysene,	WPPSI-R administered at 5 years of age	<u>Effects of high PAHs (>2.26 ng/m^3) on WPSSI-R subscales (baseline vs. fully adjusted)</u><br>Total Score: β = -3.45 (-6.63, - 0.27)* vs. β = -3.48 (-7.10, 0.15)<br>Verbal: β = -3.90 (-6.98, -0.81)* vs. β = -4.21 (-7.89, -	Baseline model: Child's sex, ethnicity, and post-natal PAH exposure; Maternal

	women from the CCCEH cohort who were registered for prenatal care at the New York Presbyterian Medical Center and Harlem Hospital by 20th week of pregnancy; 54% Black/46% Dominican; 38% maternal education < high school; 36% poverty	benzo[*b*]fluroanthene, benzo[*k*]fluroanthene, benzo[*a*]pyrene, indeno[1,2,3-*cd*]pyrene, disbenz[*a,h*]anthracene, and benzo[*g,h,i*]perylene) and determine maternal exposures at single time points during 3rd trimester<br>Ambient source of pollutants: traffic emissions		0.53)*<br>Performance: β = -1.67 (-4.89, 1.55) vs. β = -1.36 (-4.90, 2.18)<br><br><u>Interaction analyses of PAH (low vs. high PAH) exposure with neighborhood characteristics as predictors of age 5 WPSSI-R scores</u><br>*Percent Poverty*<br>Total Score: β = -0.39 (-2.89, 2.12) vs. β = -0.79 (-3.82, 2.24); p_{int} = 0.552<br>Verbal: β = 1.60 (-0.68, 3.88) vs. β = 0.33 (1.71, 2.36); p_{int} = 0.344<br>Performance: β = -2.02 (-4.72, 0.68) vs. β = -2.15 (-5.87, 1.57); p_{int} = 0.669<br><br>*Percent < high school education*<br>Total Score: β = -1.66 (-3.75, 0.44) vs. β = -0.81 (-2.32, 0.70); p_{int} = 0.014*<br>Verbal: β = 0.56 (-1.40, 2.52) vs. β = 0.26 (-0.83, 1.35); p_{int} = 0.207<br>Performance: β = -3.16 (-6.03, -0.29)* vs. β = -1.84 (4.02, 0.34); p_{int} = 0.026*<br><br>*Percent low English proficiency*<br>Total Score: β = -3.23 (-4.77, -1.69)* vs. β = -1.69 (-2.64, -0.73)*; p_{int} = 0.006*<br>Verbal: β = -1.90 (-4.36, 0.56)* vs. β = -0.51 (-1.30, 0.28)*; p_{int} = 0.048*<br>Performance: β = -3.29 (-6.97, 0.40)* vs. β = -2.52 (-4.08, -0.95)*; p_{int} = 0.003*<br><br>*Percent inadequate plumbing*<br>Total Score: β = -0.17 (-2.27, 1.93) vs. β = 0.88 (-1.08, 2.84); p_{int} = 0.145<br>Verbal: β = 1.24 (-1.44, 3.92) vs. β = 0.70 (-1.55, 2.95); p_{int} = 0.716<br>Performance: β = -1.14 (-2.95, 0.67) vs. β = 0.74 (-1.36, 2.83); p_{int} = 0.022*	education and IQ; ETS in the home, quality of caretaking environment, and household English language exposure<br><br>Fully adjusted includes household variables (building dilapidation index) and neighborhood characteristics (poverty, education, low English proficiency, and inadequate plumbing)
Jedrychowski et al. (2014) (6)<br><br>Prospective	170 children of white, healthy, nonsmoking pregnant women > 18 years old recruited between November 2000 and	Collected umbilical cord blood at time of delivery and analyzed using HPLC/fluorescence methods to measure B[a]P-DNA adducts	WISC-R administered at 7 years of age to assess verbal and performance IQs, and the difference of the	<u>Effects of PAH-DNA adducts (ln-transformed) on dichotomized DepVIQ (cutoff of 22 points)</u><br>RR = 3.00 (1.32, 6.79)*	Child's gender, post-natal indoor PAH exposure, and birth season (0 = summer; 1 = winter); Maternal education, parity (0

	March 2003 in Krakow, Poland were followed from in utero until age 7; 100% Caucasian; maternal education 15.6+2.8 years of schooling	(proxy for PAH-DNA adducts)<br>Ambient source of pollutants: traffic and industrial/residential coal burning emissions	two was taken to calculate the depressed verbal IQ index (DepVIQ) whereby DepVIQ scores ≥22 points (90th percentile) indicated cognitive dysfunction		= 1st childbearing, 1 = 2 or more), and breastfeeding practice
Harris et al. (2015) (7)<br><br>Prospective	1,109 newborns of mothers' from Project Viva who were recruited 1999-2002 at eight locations of Atrius Harvard Vanguard Medical Associates (a group practice in urban and suburban eastern Massachusetts) and were followed until mid-childhood (average 8 years); 16% Black; 32% maternal/37% paternal education <college; 13% household income ≤$40,000	Residential exposure to black carbon (BC) and $PM_{2.5}$ during 3rd trimester, at birth and date of cognitive assessment predicted using a spatiotemporal LUR model<br>Ambient source of pollutant: traffic emissions for BC plus regional sources for $PM_{2.5}$	KBIT-2 administered at 6.6-10.9 years of age	<u>Effects of air pollution (per IQR increase) on mean differences (95% CIs) in IQ scores</u><br>*BC, 3rd trimester (per 0.32 μg/m^3)*<br>Verbal IQ: 0.2 (-0.9, 1.3)<br>Nonverbal IQ: 1.3 (-0.2, 2.7)<br><br>*BC , Birth-Age 6 (per 0.22 μg/m^3)*<br>Verbal IQ: 0.9 (-0.4, 2.2)<br>Nonverbal IQ: 1.7 (0.1, 3.4)<br><br>*BC, Year before cognitive test (per 0.20 μg/m^3)*<br>Verbal IQ: 1.1 (-0.2, 2.4)<br>Nonverbal IQ: 0.7 (-0.9, 2.4)<br><br>*$PM_{2.5}$, 3rd trimester (per 3.8 μg/m^3)*<br>Verbal IQ: -0.2 (-1.4, 1.1)<br>Nonverbal IQ: -0.2 (-1.8, 1.4)<br><br>*$PM_{2.5}$, Birth-Age 6 (per 2.1 μg/m^3)*<br>Verbal IQ: 0.7 (-0.4, 1.7)<br>Nonverbal IQ: 1.1 (-0.2, 2.5)<br><br>*$PM_{2.5}$, Year before cognitive test (per 2.5 μg/m^3)*<br>Verbal IQ: 1.1 (0.0, 2.2)<br>Nonverbal IQ: 0.7 (-0.8, 2.1)<br><br>Did not observe consistent patterns of effect measure modification by sex (data was not shown)	Child's age, sex, breastfeeding duration, early-childhood blood lead exposure; Mother's age, parity, race-ethnicity, education, IQ, marital/cohabitation status, alcohol use during pregnancy, and blood lead, smoking, and secondhand smoke exposure; Father's education; Household income, home caretaking environment, gas stove, and census tract median income
Porta et al. (2015) (8)<br><br>Prospective	465 newborns of mother's from the Gene and Environment Prospective Study on	Residential exposure to NO_2, PM_{coarse}, and $PM_{2.5}$, and $PM_{2.5\ absorbance}$ at birth was predicted using a LUR model	WISC-III (Wechsler Intelligence Scale for Children)	<u>Effects of air pollutants (per IQR increase) on WISC-III subscales:</u><br>*NO_2 (per 10 μg/m^3)*<br>Full-scale IQ: β = -1.1 (-2.3, 0.10)<br>Verbal IQ: β = -1.4 (-2.6, -0.20)*	Child's gender, age at cognitive test, and number of siblings; Maternal and paternal

	Infancy in Rome, Italy who were enrolled at delivery in two large obstetric hospitals in 2003-2004 followed until 8-years of age; 60% maternal/58% paternal education ≤secondary school; 16% area-based SES low	Ambient source of pollutant: traffic emissions plus local/regional sources	administered at 7 years of age	Performance IQ: β = -0.58 (-1.9, 0.73)<br>Verbal comprehension index: β = -1.4 (-2.7, -0.20)*<br>Perceptual organization index: β = -0.48 (-1.8, 0.83)<br>Freedom from distractibility index: β = -1.2 (-2.5, 0.01)<br>Processing speed index: β = -0.17 (-1.5, 1.1)<br><br>*PM Coarse (per 5 $\mu g/m^3$)*<br>Full-scale IQ: β = -1.1 (-2.8, 0.50)<br>Verbal IQ: β = -0.59 (-2.2, 1.0)<br>Performance IQ: β = -1.4 (-3.2, 0.32)<br>Verbal comprehension index: β = -0.76 (-2.5, 0.93)<br>Perceptual organization index: β = -1.1 (-2.9, 0.63)<br>Freedom from distractibility index: β = -0.40 (-2.1, 1.3)<br>Processing speed index: β = -1.4 (-3.1, 0.37)<br><br>*$PM_{2.5}$ (per 10 $\mu g/m^3$)*<br>Full-scale IQ: β = -1.9 (-7.9, 4.1)<br>Verbal IQ: β = 0.44 (-5.5, 6.4)<br>Performance IQ: β = -4.1 (-3.4, 1.2)<br>Verbal comprehension index: β = -0.23 (-6.4, 6.0)<br>Perceptual organization index: β = -3.1 (-9.5, 3.4)<br>Freedom from distractibility index: β = 0.17 (-5.9, 6.3)<br>Processing speed index: β = -4.0 (-10, 2.4)<br><br>*$PM_{2.5\ Absorbance}$ (per $10^{-5}/m$)*<br>Full-scale IQ: β = -0.49 (-2.6, 1.6)<br>Verbal IQ: β = 0.07 (-2.0, 2.2)<br>Performance IQ: β = -1.1 (-3.4, 1.2)<br>Verbal comprehension index: β = 0.27 (-1.9, 2.5)<br>Perceptual organization index: β = -0.61 (-2.9, 1.7)<br>Freedom from distractibility index: β = -0.90 (-3.1, 1.3)<br>Processing speed index: β = -1.1 (-3.4, 1.2)	educational level, socioeconomic index at birth, maternal age at delivery, and maternal smoking during pregnancy; Psychologist who administered cognitive test and inversely weighted for the probability of participation at baseline and follow-up.
Vishnevetsky et al. (2015) (9)<br><br>Prospective	276 children of nonsmoking Dominican and African-American women (18-35 years old) residing in Washington Heights, Harlem, or the South Bronx in NY from the CCCEH cohort were recruited 1998-	Collected umbilical cord blood at time of delivery and analyzed using HPLC/fluorescence methods to measure B[a]P-DNA adducts (proxy for PAH-DNA adducts). PAH metabolites measured in	WISC-IV administered at 7 years of age	<u>Effects of prenatal PAH-DNA adducts (high: > 0.25 adducts per 10 vs. low: < 0.25 adducts per 10) exposure on IQ:</u><br>Full-scale IQ: β = -3.45 (-6.35, -0.55)*<br>Processing Speed: β = -3.72 (-7.28, -0.17)*<br>Perceptual reasoning: β = -3.02 (-6.30, 0.26)<br>(Data not shown for the rest of the subscales)<br><br><u>Interaction analyses of PAH-DNA adducts with material hardship as predictors of IQ</u><br>*Prenatal material hardship (low vs. high)*	Child's sex and ethnicity; Maternal environmental tobacco smoke exposure during pregnancy, education and intelligence; Home caretaking environment

	2006 through local prenatal care clinics, and monitored in utero until 7 years old; 38% African American; 37% maternal education ≤high school	spot urine collected at 5 years of age.		Full-scale IQ: $\beta = -1.79$ (-5.50, 1.93) vs. $\beta = -5.81$ (-10.35, -1.26)*; $\beta_{int} = -4.66$ (-10.43, 1.11)<br>Verbal comprehension: $\beta = -1.08$ (-4.29, 2.14) vs. $\beta = -3.36$ (-7.61, 0.90); $\beta_{int} = -2.39$ (-7.57, 2.80)<br>Processing speed: $\beta = -3.59$ (-8.21, 1.02) vs. $\beta = -4.17$ (-9.75, 1.41); $\beta_{int} = -0.97$ (-8.09, 6.16)<br>Perceptual reasoning: $\beta = -1.45$ (-5.86, 2.95) vs. $\beta = -5.44$ (-10.27, -0.61)*; $\beta_{int} = -4.66$ (-11.20, 1.89)<br>Working memory: $\beta = 0.57$ (-3.70, 4.85) vs. $\beta = -6.67$ (-11.38, -1.95)*; $\beta_{int} = -8.07$ (-14.48, -1.66)*<br><br>*Non-recurrent material hardship vs. Recurrent material hardship*<br>Full-scale IQ: $\beta = -1.32$ (-4.97, 2.33) vs. $\beta = -6.63$ (-11.28, -1.98)*; $\beta_{int} = -5.59$ (-11.37, 0.20)<br>Verbal comprehension: $\beta = -0.79$ (-3.98, 2.40) vs. $\beta = -4.21$ (-8.51, 0.09); $\beta_{int} = -3.13$ (-8.32, 2.07)<br>Processing speed: $\beta = -3.46$ (-7.71, 0.79) vs. $\beta = -4.02$ (-10.13, 2.09); $\beta_{int} = -0.83$ (-7.97, 6.30)<br>Perceptual reasoning: $\beta = -1.29$ (-5.55, 2.96) vs. $\beta = -5.66$ (-10.71, -0.61)*; $\beta_{int} = -4.74$ (-11.32, 1.85)<br>Working memory: $\beta = 1.24$ (-3.13, 5.60) vs. $\beta = -8.06$ (-12.49, -3.63)*; $\beta_{int} = -9.82$ (-16.22, -3.42)*	
Peterson et al. (2015) (10)<br><br>Cross-sectional	40 children of nonsmoking Dominican and African-American women (18-35 years old) residing in Washington Heights, Harlem, or the South Bronx in NY from the CCCEH cohort were recruited 1998-2006 through local	Personal air monitors to measure 8 airborne PAHs (benz[*a*]anthracene, chrysene, benzo[*b*]fluroanthene, benzo[*k*]fluroanthene, benzo[*a*]pyrene, indeno[1,2,3-*cd*]pyrene, disbenz[*a,h*]anthracene, and benzo[*g,h,i*]perylene)	WISC-IV administered at 7-9 years of age	<u>Effects of prenatal PAH exposure on processing speed</u><br>Significantly associated with low processing speed<br><br><u>Effects of postnatal PAH exposure on processing speed</u><br>No association with processing speed	Child's age and sex

	prenatal care clinics, and monitored in utero until 7-9 years old; 28% African American/73% Dominican; maternal education 12.1+2.0 years of schooling; 68% income <$20,000	and determine maternal exposures over 48-hr period during 3rd trimester<br><br>Ambient source of pollutants:<br>traffic emissions<br><br>PAH metabolites measured in spot urine collected at 5 years of age.<br>Source of pollutants: traffic emissions, dietary and dermal exposure			
Chiu et al. (2016) (11)<br><br>Prospective	267 newborns of English- or Spanish-speaking mother's (≥18 years) from the Asthma Coalition on Community, Environment and Social Stress project who were recruited 2002-2007 from Brigham & Women's Hospital, Boston Medical Center, and affiliated community health centers; 25% Black/60% Hispanic; 68% maternal education ≤12 years	Residential exposure to $PM_{2.5}$ during pregnancy was estimated using a spatiotemporal prediction model<br><br>Ambient source of pollutants:<br>Traffic emissions plus regional sources	WISC-IV administered at 6.5 ± 0.98 years of age	Effects of $PM_{2.5}$ (per IQR [10 μg/m^3] increase) exposure over gestation on full-scale IQ<br>All: no significant association<br>Boys, 31-38 weeks: β = ~ -2 (-3, -0.5)<br>Girls: no significant association<br><br>Effects of $PM_{2.5}$ (per unit increase) averaged across sensitive window on full-scale IQ<br>Boys: β = ~ -1.5 (-2, -0.5)<br>Girls: β = ~ 1.1 (-1.5, 1.5)<br><br>Effects of $PM_{2.5}$ (per unit increase) averaged over entire pregnancy on full-scale IQ<br>Boys: β = ~ -1.5 (-3, 1.2)<br>Girls: β = ~ -1.1 (-2, 2)	Child's sex; Maternal age, race, education, prenatal/postnatal smoking, parity, and blood lead level

References

1. Suglia SF, Gryparis A, Wright RO, Schwartz J, Wright RJ. Association of black carbon with cognition among children in a prospective birth cohort study. Am J Epidemiol. 2008;167(3):280-6.
2. Perera FP, Li Z, Whyatt R, Hoepner L, Wang S, Camann D, et al. Prenatal airborne polycyclic aromatic hydrocarbon exposure and child IQ at age 5 years. Pediatrics. 2009;124(2):e195-202.
3. Edwards SC, Jedrychowski W, Butscher M, Camann D, Kieltyka A, Mroz E, et al. Prenatal exposure to airborne polycyclic aromatic hydrocarbons and children's intelligence at 5 years of age in a prospective cohort study in Poland. Environmental health perspectives. 2010;118(9):1326-31.
4. Perera F, Li TY, Lin C, Tang D. Effects of prenatal polycyclic aromatic hydrocarbon exposure and environmental tobacco smoke on child IQ in a Chinese cohort. Environ Res. 2012.
5. Lovasi GS, Eldred-Skemp N, Quinn JW, Chang HW, Rauh VA, Rundle A, et al. Neighborhood Social Context and Individual Polycyclic Aromatic Hydrocarbon Exposures Associated with Child Cognitive Test Scores. J Child Fam Stud. 2014;23(5):785-99.
6. Jedrychowski WA, Perera FP, Camann D, Spengler J, Butscher M, Mroz E, et al. Prenatal exposure to polycyclic aromatic hydrocarbons and cognitive dysfunction in children. Environmental science and pollution research international. 2014.
7. Harris MH, Gold DR, Rifas-Shiman SL, Melly SJ, Zanobetti A, Coull BA, et al. Prenatal and Childhood Traffic-Related Pollution Exposure and Childhood Cognition in the Project Viva Cohort (Massachusetts, USA). Environmental health perspectives. 2015;123(10):1072-8.
8. Porta D, Narduzzi S, Badaloni C, Bucci S, Cesaroni G, Colelli V, et al. Air Pollution and Cognitive Development at Age 7 in a Prospective Italian Birth Cohort. Epidemiology (Cambridge, Mass). 2016;27(2):228-36.
9. Vishnevetsky J, Tang D, Chang HW, Roen EL, Wang Y, Rauh V, et al. Combined effects of prenatal polycyclic aromatic hydrocarbons and material hardship on child IQ. Neurotoxicology and teratology. 2015;49:74-80.
10. Peterson BS, Rauh VA, Bansal R, Hao X, Toth Z, Nati G, et al. Effects of prenatal exposure to air pollutants (polycyclic aromatic hydrocarbons) on the development of brain white matter, cognition, and behavior in later childhood. JAMA psychiatry. 2015;72(6):531-40.
11. Chiu YH, Hsu HH, Coull BA, Bellinger DC, Kloog I, Schwartz J, et al. Prenatal particulate air pollution and neurodevelopment in urban children: Examining sensitive windows and sex-specific associations. Environment international. 2016;87:56-65.
