# Supplementary material for: Socioeconomic disparities and sexual dimorphism in neurotoxic effects of ambient fine particles on youth IQ: A longitudinal analysis
Source: PLoS One. 2017 Dec 5;12(12):e0188731. doi: 10.1371/journal.pone.0188731 (PMC5716576; doi:10.1371/journal.pone.0188731)
Supplement: S1 Table — (PDF) [file pone.0188731.s004.pdf]

S1 Table. Descriptive statistics of major demographic characteristics, $PM_{2.5}$ 1-year preceding and IQ scores of three sub-cohorts

		Tested at both periods (N=380)	Tested during pre-/ early-adolescence only (N=810)	Tested during emerging adulthood only (N=170)	p-value*
**VIQ**					
	Pre-/Early-Adolescence	103.92 ± 16.79	100.55 ± 18.36	---	0.0025
	Emerging Adulthood	104.69 ± 15.20	---	103.96 ± 17.74	0.6189
**PIQ**					
	Pre-/Early-Adolescence	101.67 ± 16.57	99.58 ± 17.28	---	0.0488
	Emerging Adulthood	103.14 ± 15.80	---	101.74 ± 16.49	0.3444
**Family SES**					
	Pre-/Early-Adolescence	44.56 ± 12.32	42.04 ± 12.01	---	0.4924
	Emerging Adulthood	44.11 ± 15.87	---	44.50 ± 16.29	0.8375
**$PM_{2.5}$ 1-year**					
	Pre-/Early-Adolescence	20.28 ± 2.82	20.59 ± 2.53	---	0.0638
	Emerging Adulthood	12.52 ± 2.85	---	12.69 ± 1.87	0.3951
**Gender**					0.4281
	Male	177 (46.58%)	405 (50.00%)	88 (51.76%)	
	Female	203 (53.42%)	405 (50.00%)	82 (48.24%)	
**Race/Ethnicity**					0.1364
	White	99 (26.05%)	215 (26.54%)	64 (37.65%)	
	Hispanic	141 (37.11%)	310 (38.27%)	53 (31.18%)	
	Black	51 (13.42%)	117 (14.44%)	20 (11.76%)	
	Asian	17 (4.47%)	37 (4.57%)	4 (2.35%)	
	Mixed	72 (18.95%)	131 (16.17%)	29 (17.06%)	

*P-value for ANOVA test comparing means of continuous population characteristics; P-value for Pearson χ^2 test comparing the distribution of categorical population characteristics across three sub-cohorts.
