# Supplementary material for: Socioeconomic disparities and sexual dimorphism in neurotoxic effects of ambient fine particles on youth IQ: A longitudinal analysis
Source: PLoS One. 2017 Dec 5;12(12):e0188731. doi: 10.1371/journal.pone.0188731 (PMC5716576; doi:10.1371/journal.pone.0188731)
Supplement: S2 Table — (PDF) [file pone.0188731.s005.pdf]

S2 Table. Population Characteristics at Baseline in Relation to Levels of Verbal IQ

Population Characteristics	N†	Quartile of Verbal IQ				
		40-90 Median=82	91-102 Median=97	103-114 Median=108	115-155 Median=123	
	1360	(N=349)	(N=339)	(N=349)	(N=323)	p-value*
**Age**	1360	10.64 ± 2.89	10.47 ± 2.78	10.83 ± 3.26	11.09 ± 3.57	0.0719
**Gender**						0.2057
Male	690	160 (23.19%)	176 (25.51%)	185 (26.81%)	169 (24.49%)	
Female	670	189 (28.21%)	163 (24.33%)	164 (24.48%)	154 (22.99%)	
**Ethnicity**						<0.0001
Caucasian	378	37 (9.79%)	49 (12.96%)	107 (28.31%)	185 (48.94%)	
Hispanic	504	181 (35.91%)	167 (33.13%)	103 (20.44%)	53 (10.52%)	
Black	188	65 (34.57%)	49 (26.06%)	51 (27.13%)	23 (12.23%)	
Asian	58	12 (20.69%)	18 (31.03%)	19 (32.76%)	9 (15.52%)	
Other or Mixed	232	54 (23.28%)	56 (24.14%)	69 (29.74%)	53 (22.84%)	
**Household socioeconomic status**	1360	36.35 ± 9.91	39.7 ± 11.11	44.44 ± 10.99	48.99 ± 10.05	<0.0001
**Neighborhood socioeconomic status**	1360	-0.43 ± 0.76	-0.22 ± 0.76	0.02 ± 0.95	0.42 ± 1.17	<0.0001
**Neighborhood quality¶**	1344	29.87 ± 11.97	27.43 ± 10.06	27.59 ± 10.20	26.41 ± 8.81	0.0002
**Maternal smoking during pregnancy**						<0.0001
No	1216	290 (23.85%)	306 (25.16%)	320 (26.32%)	300 (24.67%)	
Yes	84	38 (45.24%)	24 (28.57%)	12 (14.29%)	10 (11.9%)	
**Parental WJ Score – Letter Word**	1099	53.15 ± 8.58	53.99 ± 7.16	55.72 ± 7.93	57.55 ± 7.83	<0.0001
**Parental WJ Score – Word Attack**	1099	22.01 ± 5.99	23.20 ± 5.16	23.89 ± 4.89	25.41 ± 3.87	<0.0001
**Parental Stress**	1346	34.21 ± 8.62	32.03 ± 7.90	32.14 ± 8.36	29.83 ± 8.08	<0.0001
**NDVI 1-year prior in 1000 meter area**	1360	0.29 ± 0.07	0.31 ± 0.07	0.33 ± 0.07	0.36 ± 0.09	<0.0001
**Traffic density in 300 meter area**	1360	91.61 ± 148.85	92.07 ± 156.97	86.10 ± 136.61	65.41 ± 100.29	0.0429
**Temperature 1-year prior (°C)**	1360	17.45 ± 0.71	17.42 ± 0.74	17.47 ± 0.70	17.40 ± 0.75	0.5738
**Relative humidity 1-year prior (%)**	1360	62.22 ± 5.93	61.89 ± 6.66	61.19 ± 6.45	60.93 ± 6.23	0.0281
**Total annual NOx (ppb)**	1360	31.99 ± 19.72	32.25 ± 23.06	28.88 ± 23.09	25.50 ± 18.69	<0.0001

†Total number of subjects decreases slightly due to missing values; ¶ Higher score represented a more negative perception of neighborhood quality.
*P-value from the ANOVA test comparing means of continuous variables or Pearson χ^2 test comparing the distribution of VIQ across categorical variables across the quartile of outcome variable

.
