# Supplementary material for: Socioeconomic disparities and sexual dimorphism in neurotoxic effects of ambient fine particles on youth IQ: A longitudinal analysis
Source: PLoS One. 2017 Dec 5;12(12):e0188731. doi: 10.1371/journal.pone.0188731 (PMC5716576; doi:10.1371/journal.pone.0188731)
Supplement: S3 Table — (PDF) [file pone.0188731.s006.pdf]

S3 Table. Population Characteristics at Baseline in Relation to Levels of Performance IQ

**Population Characteristics**	**N†**	**Quartile of Performance IQ**				
		**44-90**	**91-100**	**101-113**	**114-148**	
		**Median=81**	**Median=96**	**Median=107**	**Median=121**	
	**1360**	**(N=358)**	**(N=335)**	**(N=353)**	**(N=314)**	**p-value***
**Age**	1360	10.62 ± 2.93	10.47 ± 2.86	10.87 ± 3.30	11.09 ± 3.42	0.0594
**Gender**						0.2090
Male	690	194 (28.12%)	168 (24.35%)	183 (26.52%)	145 (21.01%)	
Female	670	164 (24.48%)	167 (24.93%)	170 (25.37%)	169 (25.22%)	
**Ethnicity**						<0.0001
Caucasian	378	45 (11.9%)	86 (22.75%)	112 (29.63%)	135 (35.71%)	
Hispanic	504	158 (31.35%)	133 (26.39%)	132 (26.19%)	81 (16.07%)	
Black	188	85 (45.21%)	52 (27.66%)	38 (20.21%)	13 (6.91%)	
Asian	58	7 (12.07%)	13 (22.41%)	12 (20.69%)	26 (44.83%)	
Other or Mixed	232	63 (27.16%)	51 (21.98%)	59 (25.43%)	59 (25.43%)	
**Household socioeconomic status**	1360	38.15 ± 10.42	41.86 ± 10.96	43.1 ± 11.53	46.45 ± 11.81	<0.0001
**Neighborhood socioeconomic status**	1360	-0.40 ± 0.85	-0.10 ± 0.92	0.03 ± 0.96	0.25 ± 1.05	<0.0001
**Neighborhood quality¶**	1344	28.92 ± 11.98	27.77 ± 10.65	27.97 ± 9.62	26.62 ± 8.91	0.0412
**Maternal smoking during pregnancy**						0.0963
No	1216	301 (24.75%)	300 (24.67%)	327 (26.89%)	288 (23.68%)	
Yes	84	30 (35.71%)	22 (26.19%)	16 (19.05%)	16 (19.05%)	
**Parental WJ Score – Letter Word**	1099	53.00 ± 7.46	55.36 ± 7.97	55.68 ± 8.35	56.44 ± 8.04	<0.0001
**Parental WJ Score – Word Attack**	1099	22.22 ± 6.20	23.53 ± 4.98	23.78 ± 4.86	25.11 ± 3.88	<0.0001
**Parental Stress**	1346	32.96 ± 8.64	32.54 ± 8.53	31.97 ± 8.57	30.75 ± 7.57	0.0051
**NDVI 1-year prior in 1000 meter area**	1360	0.30 ± 0.07	0.32 ± 0.08	0.32 ± 0.08	0.35 ± 0.08	<0.0001
**Traffic density in 300 meter area**	1360	81.12 ± 134.91	86.09 ± 141.07	94.78 ± 157.72	73.31 ± 112.29	0.2345
**Temperature 1-year prior (°C)**	1360	17.38 ± 0.72	17.47 ± 0.70	17.44 ± 0.75	17.46 ± 0.73	0.3226
**Relative humidity 1-year prior (%)**	1360	62.31 ± 6.24	61.88 ± 6.31	61.24 ± 6.28	60.76 ± 6.45	0.0079
**Total annual NOx (ppb)**	1360	32.42 ± 21.94	30.07 ± 21.35	28.47 ± 21.88	27.66 ± 20.06	0.0194

†Total number of subjects decreases slightly due to missing values; ¶ Higher score represented a more negative perception of neighborhood quality.
*P-value from the ANOVA test comparing means of continuous variables or the Pearson χ^2 test comparing the distribution of categorical variables across the quartile of outcome variable.

.
