# Supplementary material for: Socioeconomic disparities and sexual dimorphism in neurotoxic effects of ambient fine particles on youth IQ: A longitudinal analysis
Source: PLoS One. 2017 Dec 5;12(12):e0188731. doi: 10.1371/journal.pone.0188731 (PMC5716576; doi:10.1371/journal.pone.0188731)
Supplement: S4 Table — (PDF) [file pone.0188731.s007.pdf]

S4 Table. Associations between total annual NO_x and subscales of IQ

Models	N[†]	Full-Scale IQ β (95% CI) [#]	VIQ β (95% CI) [#]	PIQ β (95% CI) [#]
Crude Analysis	1362	-1.66 (-2.5, -0.82)*	-1.12 (-2.04, -0.20)*	-1.28 (-2.12, -0.44)*
Adjusted Model I [¶]	1093	0.26 (-0.76, 1.28)	0.43 (-0.67, 1.53)	0.09 (-0.97, 1.15)
Adjusted Model II [§]	1085	0.12 (-1.15, 1.39)	0.90 (-0.47, 2.27)	-0.26 (-1.57, 1.05)
**Sensitivity Analyses**				
Sensitivity Model I[††]	1085	0.29 (-1.00, 1.58)	0.96 (-0.43, 2.35)	-0.13 (-1.46, 1.20)
Sensitivity Model II[**]	1085	0.24 (-1.05, 1.53)	1.05 (-0.32, 2.42)	-0.23 (-1.56, 1.10)
Sensitivity Model III[##]	1042	0.12 (-1.15, 1.39)	0.93 (-0.44, 2.30)	-0.10 (-1.43, 1.23)

* $P < .05$

[†] Total number of participants differed because of missing values.

[#] Estimate reflected the change in each IQ score and the resulting 95% confidence interval per each inter-quartile range (IQR) increase in total annual NO_x.

[¶] Crude Analysis + age, gender, ethnicity, family SES and parents' cognitive abilities; age

[§] Adjusted Model I + neighborhood SES, self-reported neighborhood quality, traffic density (300m) and $PM_{2.5}$ 1-year prior to test.

[††] Adjusted Model II + temperature and relative humidity 1-year prior to test.

[**] Adjusted Model II + neighborhood greenness (1 year, 1000m).

[##] Adjusted Model II + parental stress and maternal smoking during pregnancy.
